# Early Pathologic Findings of Bronchiolitis Obliterans after Allogeneic Hematopoietic Stem Cell Transplantation: A Proposal from a Case

**DOI:** 10.1155/2012/957612

**Published:** 2012-08-22

**Authors:** Rie Nakamoto-Matsubara, Hidekazu Nishikii, Kenji Yamada, Masafumi Ito, Yuichi Hasegawa, Naoki Kurita, Naoshi Obara, Yasushi Okoshi, Kazumi Suzukawa, Yasuhisa Yokoyama, Mamiko Sakata-Yanagimoto, Masayuki Noguchi, Shigeru Chiba

**Affiliations:** ^1^Department of Hematology, Faculty of Medicine and Graduate School of Comprehensive Human Sciences, University of Tsukuba, 1-1-1 Tenoudai Tsukuba, Ibaraki 305-8575, Japan; ^2^Department of Pathology, Graduate School of Comprehensive Human Sciences, University of Tsukuba, 1-1-1 Tenoudai Tsukuba, Ibaraki 305-8575, Japan; ^3^Department of Pathology, Japanese Red Cross Nagoya Daiichi Hospital, 3-35 Michishita-cho, Nakamura, Nagoya 453-8511, Japan

## Abstract

Bronchiolitis obliterans (BO) is one of the serious, noninfectious pulmonary complications after allogeneic hematopoietic stem cell transplantation (allo-HSCT). Early diagnosis of BO is usually difficult because patients are often asymptomatic at an initial stage of the disease and pathologic findings are available mostly at the late stages. Therefore, the diagnosis of the disease is based on the pulmonary function test using the National Institute of Health consensus criteria. Here, we report a case of slowly progressive BO. A biopsy specimen at an early stage demonstrated alveolar destruction with lymphocyte infiltration in bronchial walls and mild narrowing of bronchioles without fibrosis, those were strongly indicative of initial pathologic changes of BO. Definitive BO followed, which was proven by both clinical course and autopsy. While alloreactive lymphocytes associated with chronic graft-versus-host disease are believed to initiate BO, we present a rare case that directly implies such a scenario.

## 1. Introduction

The pathogenesis of bronchiolitis obliterans (BO), a serious complication of allogeneic hematopoietic stem cell transplantation (allo-HSCT), is still unclear, whereas association with the presence of chronic graft-versus-host disease (GVHD) has been repeatedly documented [1]. BO usually develops insidiously, which hampers the opportunity of lung biopsy at an early stage. The National Institute of Health has suggested clinical diagnostic criteria based on the results of pulmonary function test (PFT) and high-resolution computed tomography (HRCT). Both of these, however, show a positive result mostly when BO is established.

Here, we report a case of slowly progressive BO that reached the end stage 10 years after allo-HSCT. Lung biopsy at the early stage of the disease demonstrated lymphocyte infiltration and narrowing of the bronchioles without fibrosis, while the results of autopsy demonstrated typical findings of end-stage BO.

## 2. Case Report

 A 27-year-old woman in third remission of acute myeloid leukemia received allo-HSCT from a 6/8 human-leukocyte-antigen- (HLA-) matched unrelated donor (HLA-C and HLA-DR mismatch) in 1999. X-ray and computed tomography (CT) of the chest and pulmonary flow test (PFT) before allo-HSCT did not reveal any abnormalities. She was not a smoker. The conditioning regimen consisted of 6-fractionated 12 Gy total body irradiation and 120 mg/kg cyclophosphamide. Cyclosporine A (CyA) and short-term methotrexate were used as a GVHD prophylaxis.

Acute GVHD of the skin was observed on day 14. Because it extended rapidly throughout the whole body, oral predonisolone (PSL) at 1 mg/kg was started. While tapering the PSL dose, chronic GVHD of the skin developed in a quiescent manner, and, thus, low-dose of PSL was continued until day 287. She then complained of dry cough and dyspnea on day 295. Serum fungal antigens and cytomegalovirus (CMV) pp65 antigenemia assay were negative. X-ray and HRCT analysis did not reveal any abnormalities. PFT indicated mild restrictive pulmonary dysfunction (FEV1.0%, 98.6%; %VC, 68.1%). Transbronchial lung biopsy (TBLB) showed mild narrowing of bronchioles and alveolar destruction with infiltration of lymphocytes in bronchial walls. There were no fibrotic lesions or severe obliteration of bronchioles (Figure 2(a)). According to the clinical course and pathological findings, we tentatively diagnosed as early stage of BO after allo-HSCT.

Although, oral PSL therapy at 1 mg/kg was resumed, her dyspnea was unchanged.

**Figure 1:**
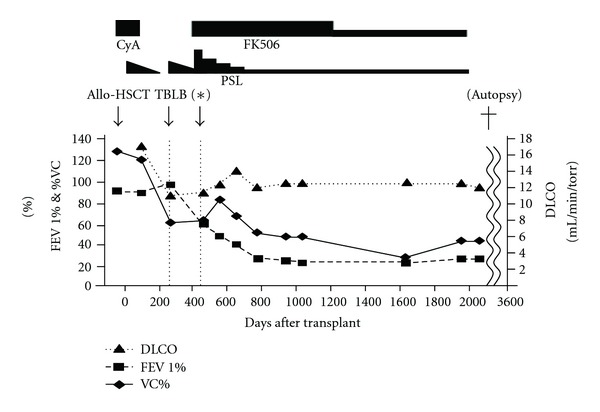
Clinical course and the results of PFT. The solid line and the dotted lines show DLCO, FEV1.0%, and %VC. (∗) The time point that diagnosis of BO was made according to the result of PFT. CyA, cyclosporine A; FK506, tacrolimus; Allo-HSCT, allogeneic hematopoietic stem cell transplantation; TBLB, transbronchial lung biopsy; PSL, predonisolone; DLCO, diffusing capacity for carbon monoxide; FEV1.0%, forced expiratory volume 1.0(sec) %; %VC, % vital vapacity.

At the period around day 480 while tapering PSL, her dyspnea worsened. PFT showed moderate to severe obstructive pulmonary dysfunction with restrictive dysfunction (FEV1.0%, 60.1%; %VC, 69.6%; Figure 1), compatible with typical BO. After the steroid pulse therapy, oral tacrolimus with PSL at 1 mg/kg was started. Although low-dose oral PSL and tacrolimus were continued, respiratory function was gradually deteriorated until day 3,000 (Figure 1). She refused the lung transplantation. Because of chronic renal dysfunction, oral tacrolimus therapy was discontinued on day 3300. On day 3600, she fell off the stairs in the house, which caused traumatic pneumothorax and died.

**Figure 2:**
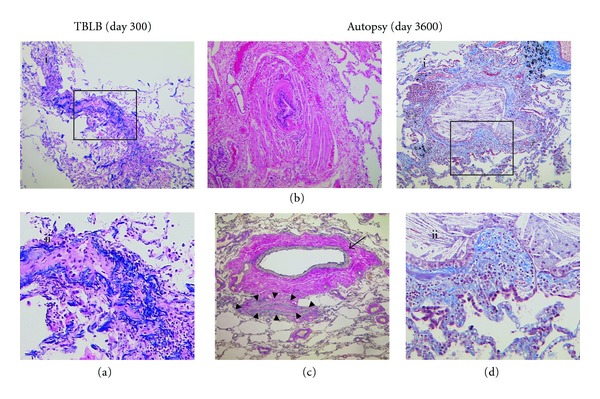
Pathological findings of the TBLB and autopsy. In the TBLB specimens, Victoria blue staining shows the narrowing of bronchioles (a-i) with infiltration of lymphocytes in bronchial walls. There are no fibrotic lesions (a-ii). In the lung tissue at autopsy, there is extensive obliteration or disappearance of bronchioles due to prominent smooth muscle hypertrophy and submucosal collagen deposition (b). Elastica van Gieson staining shows preexisting elastic fibers of the bronchiolar wall with complete fibrous obliteration, which suggests that there was the end-stage obstruction of bronchiole (arrow heads) with fibrotic tissues, surrounded by elastic fibers (black). Residual bronchial arteriole (arrow) is also observed (c). Infiltration of lymphocytes and macrophages into bronchial wall (d-ii) was occasionally observed in residual bronchioles (d-i) by Masson's Trichrome staining (blue; collagen layer in bronchial wall).

An autopsy revealed that there was extensive obliteration or disappearance of bronchioles (Figures 2(b) and 2(c)). Residual bronchioles showed luminal narrowing due to submucosal collagen deposition and smooth muscle hypertrophy of bronchiolar walls (Figure 2(b)). These findings were compatible with those at the end stage of BO [2]. Infiltration of lymphocytes in collagen layer with mild luminal narrowing, which was reminiscent of the findings observed in TBLB at the early stage, was also observed in some bronchial lumens (Figure 2(d)). There were no pathological findings of infection. These observations suggested that the destruction of bronchiole was still progressive even 10 years after diagnosis.

## 3. Discussion

Pathogenesis of BO after allo-HSCT is poorly understood. Several reports have suggested that the pathogenesis of BO is strongly associated with the presence of chronic GVHD and graft-versus-leukemia effect (GVL) [3]. Allo-reactive T cells are believed to cause the initial events of BO in the context of chronic GVHD, with very little direct evidence [1]. Infiltration of lymphocytes in the bronchial walls was rarely reported in the early stage of the disease. Biopsy specimens, mostly obtained at the later stages, demonstrate bronchiolitis involving small airways and fibrinous obliteration of the lumen of the respiratory bronchioles [4]. Infiltration of neutrophils and macrophages at the residual bronchioles was frequently observed [5]. At the end stage, the histology is characterized by peribronchiolar fibrosis and disappearance of bronchioles.

In the current case, there was infiltration of lymphocytes in bronchial walls without fibrosis, together with narrowing of bronchioles, in TBLB specimen at the clinically emerging stage of BO. At the autopsy, the residual lung tissues showed variable degrees of mature and immature fibrosis, implying typical end-stage BO. Because, from the clinical points of view, the patient's pulmonary damage progressed in a seamless manner, pathological findings at early and end stages in this case were considered as the results of the same disease. These findings imply that initiation of immune reaction of BO after allo-HSCT was mediated by donor lymphocytes.

Consequently, we suggest that lymphocyte infiltration in bronchial walls without fibrosis and mild narrowing of bronchioles should be early pathological findings of BO after allo-HSCT. These findings are useful for early diagnosis of BO and provide a clue to understand the mechanism of them.
